# ASO Author Reflections: Benchmarks in Liver Resection for Intrahepatic Cholangiocarcinoma

**DOI:** 10.1245/s10434-024-14978-7

**Published:** 2024-01-23

**Authors:** Laura Alaimo, Yutaka Endo, Timothy M. Pawlik

**Affiliations:** 1 Department of Surgery, Division of Surgical Oncology, The Ohio State University, Wexner Medical Center and James Comprehensive Cancer Center, Columbus, OH USA; 2 Department of Surgery, University of Verona, Verona, Italy

## Past

Resection of intrahepatic cholangiocarcinoma (ICC) often requires complex liver surgery, which can be associated with increased risk of complications and mortality. Surgical approaches to ICC can also differ in terms of utilization of lymphadenectomy and minimally invasive techniques.^1^ In addition, there are no established reference values to assess the quality of outcomes related to major liver surgery for ICC. The lack of standardized data at various centers can make comparisons of quality among different centers not possible. Benchmarking is a methodology that establishes quality measures by comparing clinical outcomes against key performance indicators.^2,3^ The current study was important because we identified clinically relevant perioperative outcomes associated with curative-intent surgery for ICC and established benchmark values that can be applied across a heterogeneous population of centers.^4^

## Present

Among the 14 participating centers (USA/Canada: *n* = 5, Europe: *n* = 6, Asia: *n* = 3), 1193 patients underwent surgery for ICC. The benchmark group included 600 (50.3%) patients with no preoperative jaundice, ASA class < 3, body mass index < 35 km/m^2^, and no need for bile duct or vascular resection. Intraoperative benchmark values included ≥ 3 lymph nodes retrieved during lymphadenectomy, estimated blood loss (EBL) ≤ 600 mL, perioperative blood transfusion rate ≤ 42.9%, and operative time ≤ 339 min. Postoperative benchmarks included achievement of textbook oncologic outcome (TOO) ≥ 59.3%, positive resection margins ≤ 27.5%, Clavien–Dindo III or more complications ≤ 14.3%, 30-day readmission ≤ 3.6%, and 90-day mortality ≤ 4.8%. Notably, outcomes such as the number of retrieved lymph nodes, operation time, EBL, and incidence of transfusion varied widely across institutions (number of retrieved nodes: 0–9; operative time: 108.0–521.0 min; EBL: 200–895 mL; transfusion: 14.3–64.3%). Similarly, length of stay (5.0–18.0 days), as well as incidence of margin-positive resection (0–40.0%), severe complications (0–42.9%), and subsequent TOO achievement (45.7–100%) differed markedly among institutions.

## Future

Benchmarks may serve as a valuable tool to compare outcomes across diverse cohorts, enabling the evaluation of surgical performance and oncological efficacy in the management of ICC worldwide. Data from this large multi-institutional study provide reference benchmark values for major hepatic surgery among patients undergoing liver resection of ICC. These benchmarks may facilitate the comparison of outcomes among different patient cohorts, aiding in the assessment of surgical performance and oncological efficacy in surgical management of ICC across the globe. In addition, the substantial heterogeneity observed even among high-volume centers highlights the need for ongoing quality improvement measures to improve outcomes following complex hepatic operations.
